# Transmyocardial laser revascularization in treatment chronic coronary artery disease

**DOI:** 10.1186/1749-8090-8-S1-O196

**Published:** 2013-09-11

**Authors:** J Piatek, J Konstanty-Kalandyk, A Kedziora, J Sadowski

**Affiliations:** 1 Department of Cardiovacular Surgery and Transplantology, John Paul II Hospital, Cracow, Poland

## Background

Transmyocardial laser revascularization is an indirect method of treatment CAD. Many authors underline benefits from TMLR as an additional procedure for patients in whom complete revascularization is not reachable. Channels created by the laser stimulate inflammation, which results in TMLR-related neoangiogenesis.

The aim of the study was to establish the long-term follow-up after TMLR.

## Methods

95 patients admitted for elective CABG with TMLR or isolated TMLR between 1997 and 2002 (using HOLIUM:YAG laser). In 11 cases (11.6%) cases only TMLR was applied.

## Results

Perioperative mortality was of 7.4%, long-term follow-up revealed 13.3% mortality, with 50% rate of cardiac associated deaths. MACE-free rate during 10 to 15 years follow-up period amounts 63.3%. General incidence of myocardial infarct (MI) after the procedure accounted for 34.5%. The improvement of the quality of life ascertained by patients was observed in all cases. Patients were we divided into two groups based on whether LAD grafting was (56.7%) or was not (43.3%) performed. In 23.5% cases with LAD graft, TMLR was also applied to the anterior myocardium. Improvement of the quality of life assessed by the EQ-5D scale is higher in patients without LAD grafting (42.5 vs. 31.25) (p=0.215). Patients with LAD bypass graft benefited lower MI incidence and mortality in long-term follow-up (33.3% vs. 41.7% for MI incidence and 7.7% vs. 23.1% for mortality). Applying TMLR at anterior wall additionally to bypass grafting on LAD resulted in only 25% MI incidence and no deaths in long-term follow-up.

## Conclusions

This study indicates a significant effect on angina relief in patients, who received TMLR, measured by the EQ-5D scale. We believe that in patients with end-stage CAD TMLR should be applied as an additional procedure to direct revascularization. Beneficial influence of this combined procedure was confirmed by diminished incidence of MI and death in the long-term follow-up.

